# Moving to learn: Enhancing anatomy education through physical activity and video‐based instruction

**DOI:** 10.1002/ase.70095

**Published:** 2025-07-15

**Authors:** Maureen Schaefer, Libby Bradley, Nicole Geske, Nebiyat Girma, Laura Gjidoda

**Affiliations:** ^1^ Department of Radiology Michigan State University East Lansing Michigan USA; ^2^ College of Human Medicine Michigan State University East Lansing Michigan USA

**Keywords:** active learning, anatomy education, embodied cognition, experiential learning, musculoskeletal anatomy, sensorimotor learning

## Abstract

Multiple studies have demonstrated the linkage between sedentary lifestyles and adverse health outcomes, emphasizing the need to prioritize student movement and health as a part of the medical school curriculum. This qualitative study assessed the relationship between movement‐centered teaching and students' perceptions of learned content through the integration of exercise anatomy videos into a Musculoskeletal Systems first‐year Osteopathic Medicine course at a Michigan State University. One‐on‐one student interviews were conducted, and the transcribed interviews were used to identify key themes using an inductive coding process. Results demonstrated the exercise videos aided learning by associating movements or senses with learning, repetition, and time efficiency, as well as boosting mental and physical wellness by reducing stress and anxiety and incorporating physical activity. Overall, this research emphasizes the multiple benefits of incorporating movement into anatomy education.

## INTRODUCTION

The mind body connection between movement and learning has been recognized as early as the 1800s, exemplified by Thoreau's^1^ observation “Methinks that the moment my legs begin to move, my thoughts begin to flow.” Building on this insight, it is reasonable to suggest that universities should prioritize movement as a core component of education by providing the infrastructure and resources to support physical activity within the academic environment.^2^ Despite this, students continue to engage in prolonged sedentary activity driven by academic obligations such as lectures and studying.^3^, ^4^ A sedentary lifestyle not only impacts our physical health—contributing to chronic conditions like metabolic syndrome, hypertension, obesity, type 2 diabetes, and cardiovascular disease^5^, ^6^, ^7^—but also affects mental well‐being, correlating with increased levels of depression and anxiety.^8^, ^9^, ^10^, ^11^

As a result, there is growing support for universities to encourage student movement, both for health benefits and to enhance learning.^2^, ^12^, ^13^ Research has shown that incorporating short movement breaks during longer university classes reduces sedentary behavior while boosting concentration, alertness, and overall enjoyment.^12^, ^13^, ^14^ These breaks, lasting between 3 and 10 min, may involve activities such as stretching, running, walking, or calisthenics (e.g., squats). However, a significant barrier to the widespread implementation of such interventions is the discomfort many faculty members feel in leading physical activity sessions.^2^

Beyond promoting physical health, movement has been shown to enhance cognitive function through two key pathways.^15^, ^16^, ^17^ One involves the physiological changes induced by exercise, while the other focuses on improved cognitive processing when movement is integrated into learning.^18^ Both long‐term training and single bouts of exercise optimize our physiological readiness for learning. The former strengthens brain structures and enhances neural connectivity, while the latter fosters temporary improvements in alertness and concentration when synchronized with learning events. Movement‐integrated learning, however, deepens mental representations of content, leading to faster and more effective memory recall.^18^, ^19^, ^20^

In recent years, there has been growing interest in incorporating movement into anatomy education at both the undergraduate and medical school levels. Yoga, for example, has proven useful in illustrating musculoskeletal anatomy through various poses.^21^, ^22^, ^23^, ^24^ Although studies on the effectiveness of yoga‐enhanced study sessions in improving test scores are mixed,^22^, ^24^ surveys consistently report positive student perceptions.^21^, ^22^, ^23^, ^24^ For example, Sugrue et al.^24^ reported that the sessions helped students name muscle groups, understand their function, and recall relevant clinical facts. Additionally, increased levels of enjoyment and relaxation were key findings from the data. Enhanced student enjoyment has been previously demonstrated when students actively participate in learning the course materials.^25^, ^26^

An under developed area of anatomy education involves integrating it with self‐defense techniques. Stein et al.^27^ invited undergraduate students to a seminar led by police officers, where self‐defense tactics were demonstrated alongside discussions of anatomical principles. While students reported finding the session helpful, many suggested that more audience participation would improve the experience.

Despite the success of these movement‐based approaches, teaching anatomy through yoga or martial arts has not yet been widely adopted. One explanation is that many anatomy instructors lack the training to lead exercise classes, leaving them feeling unprepared to implement such methods.^2^

To address these concerns, one of the authors (Schaefer) utilized her expertise in dance, martial arts, yoga, and general exercise to develop a series of exercise anatomy videos. These videos seamlessly integrate physical activity—from subtle movements to full‐body exercises—with anatomy lessons and are available globally through the YouTube channel “Knockout Anatomy”.^28^ The purpose of this study is to assess student perceptions of learning benefits when movement is combined with anatomy education via an accessible video platform. We hypothesize that the results will align with those of previous “in‐person” movement‐based sessions, demonstrating the utility of these videos for faculty who wish to integrate physical activity into their curricula but lack the confidence to lead such sessions themselves.

## METHODS

A qualitative study was conducted to explore first‐year medical students' perceptions of the learning benefits of incorporating movement into anatomy education through instructional videos. This study received exempt approval from MSU's Institutional Review Board (Approval Number: 00001126).

### Participants

This study was conducted at Michigan State University College of Osteopathic Medicine. One hundred thirty‐eight first‐year students enrolled in the musculoskeletal systems (MSK) course during the Fall 2023 semester were invited to participate via a weekly newsletter. To be eligible for the study, students must have self‐reported as watching at least two or more of the supplemental exercise anatomy videos recommended as preparatory materials during their course. Thirty‐three eligible students responded; however, only 28 continued to schedule an interview. Of these 28 students, 18 were female and 10 were male. Participants were compensated 20 dollars for their time. By the end of the interview process, saturation of responses had been achieved.

### Exercise anatomy videos

A series of six exercise anatomy videos (Table 1) dedicated to teaching neuromusculoskeletal anatomy were utilized for this research study. While the videos were designed as assigned supplemental resources during the MSK course, each video led students through some type of physical activity while instructional content was explained to (1) get students out of their chairs and (2) help students better retain the content.

**FIGURE 1:**
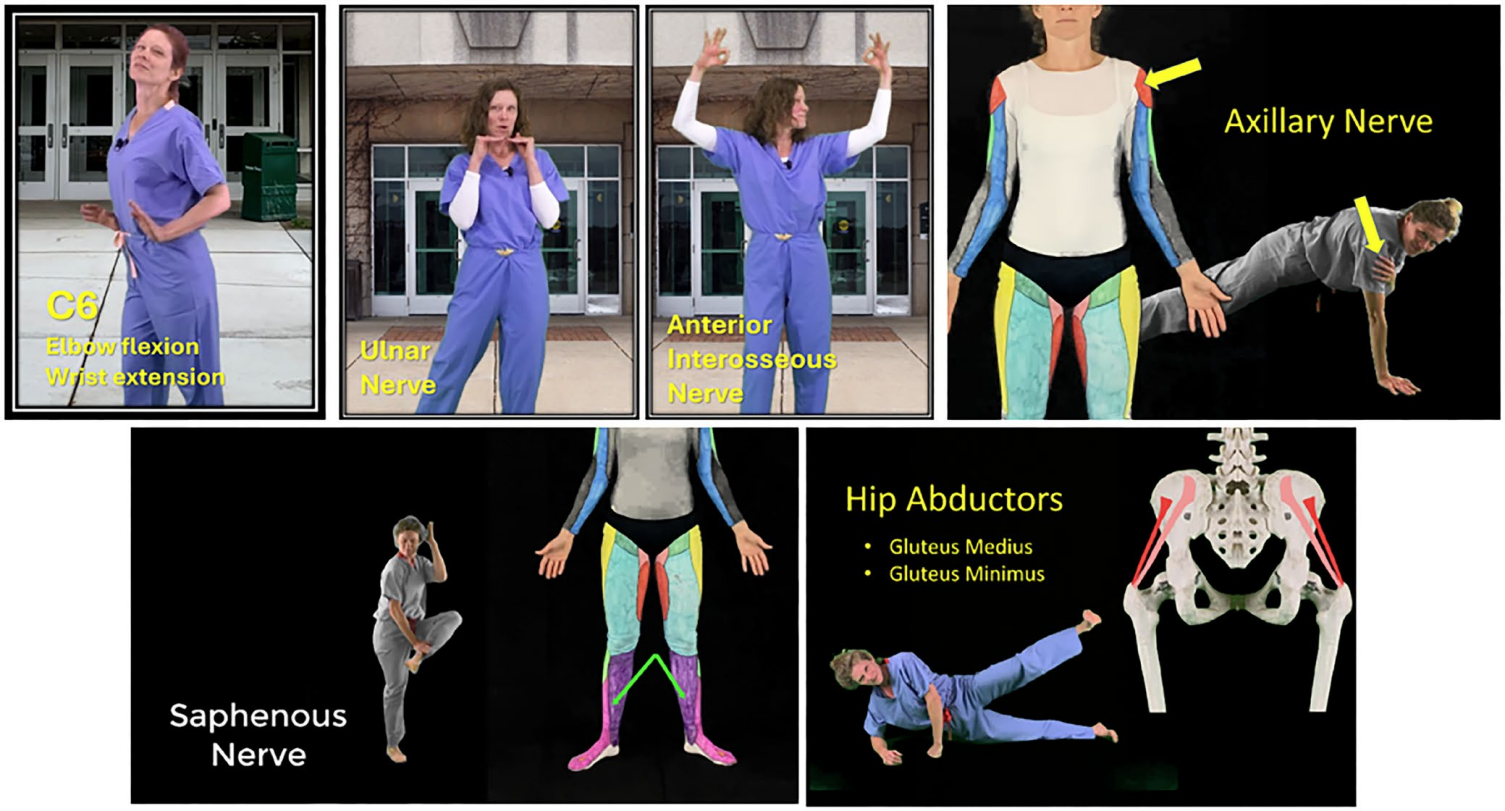
Demonstration of physical activity utilized to teach anatomy content. Moving from left to right, top to bottom. Choreographed dance move that incorporates elbow flexion and wrist extension to demonstrate the C6 myotome. Dancing the “intrinsic plus” and “OK sign” with hand gestures to remember motor functions of the ulnar and anterior interosseous nerves. Tapping the shoulder to receive tactile feedback while planking to demonstrate the sensory domain of the axillary nerve. Tapping the medial leg to receive tactile feedback while participating in aerobic‐style exercise to demonstrate the sensory domain of the saphenous nerve. Performing side lying leg lifts while discussing the hip abductors and superior gluteal nerve.

**TABLE 1:** Description of the six movement centered anatomy videos in terms of the anatomy discussed and the physical activity utilized within the teaching.

Title	Anatomy discussed	Physical activity (Figure 1)
Myotome/Dermatome Dance	Myotomes and dermatomes of upper and lower limbs	Dance movements that incorporate the actions enabled by each spinal level or touch the patches of skin innervated by each spinal level
Dance the Brachial Plexus: Part 1	Brachial plexus terminal branches	Dance movements that highlight key actions enabled by the muscles innervated by each branch
Dance the Brachial Plexus: Part 2	Brachial plexus pre and post plexus branches	
Neuromuscular Anatomy of the Lower Limb	Motor nerves of the lower limb	Performing exercises that utilize muscles innervated by the nerve that is called out
Sensory Nerves of the Lower Limb	Cutaneous innervation of the lower limb	Aerobic or calisthenic style exercises unrelated to content; each movement ends by activating the area of sensory innervation from the nerve being discussed through touching or tapping area on ground
Sensory Nerves of the Upper Limb	Cutaneous innervation of upper limb	

### Data collection

Following completion of the MSK course, students were asked to participate in a one‐on‐one semi‐structured zoom interview, lasting approximately 10–15 min. The interview consisted of open‐ended questions regarding current and past levels of physical activity, the importance of movement in their life, and how the videos made them feel and may have contributed to their learning (Table 2). Two second‐year medical students within Michigan State University's College of Human Medicine (NG and LG) conducted the interviews. Interviewers were trained in qualitative interview techniques prior to data collection. Each interview was recorded using the Zoom feature, which also transcribed the responses. NG and LG then read through the transcripts, while listening to the Zoom recording, to correct discrepancies. Students who participated in the interviews were de‐identified using a numerical progression based on the date and time of the interview.

**TABLE 2:** Script utilized for interviews.

*Background information* 1. Describe your level of activity/movement on a weekly basis. Have your activity levels changed since starting medical school? Could you comment on your level of activity/movement prior to medical school? Have the activities/movements themselves changed since beginning medical school (i.e., more walks, strength training, and yoga)? 2. Describe the level of importance of movement in your life. If there has been a change in activity/movement since medical school: Has the change in the level of activity/movement impacted your ability to focus? Have you felt an impact on the change in level of activity/movement regarding your mental health? 3. Are there any barriers in your life that prevent you from exercising or increasing your levels of activity? Have you found any ways to overcome these barriers? 4. Follow up question to the first 3: walk me through a typical study day for you. Where do you find time for movement (if not already commented on)? How do you feel during this time of movement? How do you feel after this time of movement? Do you have a current vs. ideal study day or week in terms of balancing all the tasks with movement? *Thoughts regarding videos* 5. What motivated you to watch the video(s)? 6. Can you recall which video(s) you watched? For each video that they watched, ask what was most beneficial for their learning. During the videos, did you participate or watch the video? After discussing all watched videos, ask if there was any video in particular that has stuck with/helped them. 7. Describe how you felt mentally and/or physically after participating in the video/videos. After watching these videos and/or participating, have your thoughts about movements changed? Has your activity/movement changed? Explain.

### Data coding and analysis

An inductive coding process consistent with thematic coding was used to analyze the transcripts based on the research question.^29^ Three coders (MS, NLG, and LB) read the responses of four randomly selected interviews, with each creating an initial codebook using Dedoose software (Dedoose, Los Angeles, CA: SocioCultural Research Consultants, LLC). Once complete, codes were shared, discussed, and refined to reflect everyone's contributions. The 28 interviews (including the initial four) were then coded using the refined codebook. The new set of codes from each researcher was shared once again to resolve any discrepancies in the system. Once full agreement was attained, codes were gathered into larger themes based on their overall subject matter. The larger themes were then related to the primary research questions.

## RESULTS

Students' motivation to watch the videos was driven by a desire to learn and improve their understanding of the material. Many students sought out any resource that might give them an edge, while others were particularly drawn to the movement‐based approach to learning. “I knew that MSK was a very difficult topic. I wanted to see what different resources could help me learn it because just sitting down and reading the material doesn't always help. And with a very movement‐based subject like MSK, I thought that watching the videos might be able to help (Student 14).” Student 19 shared “I thought it was an interesting way of putting our material into a physical form. I feel like I was hitting a block with the way the material was presented before, since its mostly just text. Having a video with actual movements seemed pretty beneficial to watch.”

**FIGURE 2:**
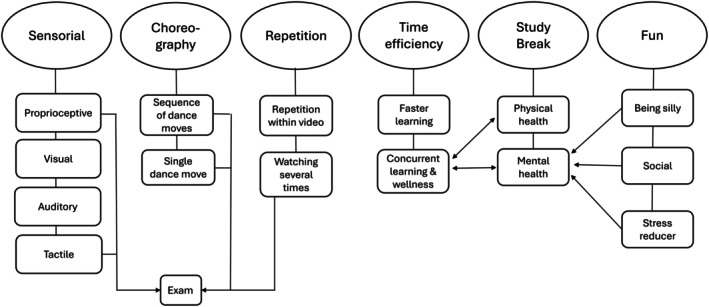
A visual depiction of the key themes and subthemes surrounding student perceptions of the learning benefits of integrating movement into anatomy education.

Following an inductive coding process, several key themes were created portraying the learning benefits of the movement‐based videos. These included linking knowledge to the senses, choreography‐driven learning, the value of repetition, time‐efficient learning, providing a break to reset for additional studying, and creating a fun learning environment. Figure 2 visually represents these themes, their subthemes, and their interconnections. Ultimately, the integration of movement and knowledge allowed students to form deeper, more meaningful connections with the material, enhancing both comprehension and memory retention. Many of these benefits were further described as assisting with their assessments. Students commented on performing various movements from the videos while taking the exam to help them remember the content.

### Sensorial experiences

Linking knowledge to the senses was found to significantly increase student connectivity with the material. The benefit of proprioceptive, visual, auditory, and, or tactile cues was mentioned in numerous excerpts. Students felt that the enhanced sensorial experience promoted a higher level of learning. “*I like to think that it (sensory input) initiates certain neurons to be firing, and it strengthens those connections*. (Student 24).”

Proprioceptive feedback gained from “doing the movement” was the most frequently cited aspect that assisted with knowledge retention. Twenty‐one excerpts by 18 students specifically mentioned physical activity as the key to creating a stronger connection with the material. This linkage was often described in terms of performing an action, such as flexing the knee, while simultaneously discussing the muscles and nerves involved in that movement. In essence, students were “doing” and “feeling” the function of the muscle group or nerve in real time. As one student noted, *“The most useful thing was the incorporation of the movement with the thing that we're learning. You know this muscle does this? Oh, if I move it, wow, there's a good, real‐life example. It's literally applicable. So, it's easier to make association and learn that way”* (Student 1). Another student reflected on how “doing the movement” helped in their lab work: *“The videos were helpful for understanding function because when I got to lab, I was able to mentally remember what I did. When we're moving and watching something, I feel like you maybe digest it a little bit better” (Student 6)*.

After watching the videos, some students described further applying the concept of learning via doing to their own physical activity regimes. *“Watching the exercises, and then having a background of weightlifting brought everything together because it was like, oh, when I do that part of my lift, that's what's firing.”* (Student 4). Another student commented that they learned to apply this type of learning experience to their running. *“Sometimes when I am running, I'm thinking about the different muscles or the different innervation sets that are utilized for each step or for each action that I take, and you know, that's information that may not have been as clear without watching these videos”* (Student 3).

Other students commented specifically on being visual learners and that seeing the movement really helped with their learning. *“I feel like I'm very much a visual learner, so to have someone demonstrate what it looks like when they're doing the action, or they're pointing it out on their body rather than just me staring at a document and trying to imagine it in my head. Seeing someone explain it to me visually helped me understand the material a lot better”* (Student 10).

Utilizing popular songs in some of the videos also led to a strong auditory connection to the material. Ten students noted that the choice of music was fundamental in helping them learn the content. Student 2 expressed that *“having a catchy song that I recognize and already remembered in my brain and then having it tied in with the stuff we were learning just kind of made it easier to click in my brain”*.

Some of the videos utilized the sensation of touch to help remember sensory domains of various cutaneous nerves or dermatomes. Participants were directed to physically touch the area of skin innervated by the nerve being discussed to further layer tactile information onto their knowledge base. Nine students noted that stimulating the relevant sensory domain was significantly useful to their learning. Student 7 commented that *“the most beneficial were the times when the cutaneous distribution of the nerve that was called out was being slapped on the ground. I was able to feel that portion of my arm hitting the ground trying to help memorize that. Then on the exam I was kind of feeling that in my head.”* Rather than just thinking about it, Student 24 commented on performing the action during the exam, stating that *“When I was in the exam, I'm tapping on myself [sensory domains] trying to remember and it becomes a lot easier than just rote memorization, which I have some struggles with.”*

### Choreography driven learning

A few students specified that the choreography itself impacted their learning. Learning the movements was easier to remember than the anatomical fact. The choreography could then be used as a platform to scaffold the anatomy. This was particularly true for the myotome dance. Students could primarily memorize the sequence of movements, and then layer the sequence of spinal levels on top of that information. *“My favorite part was that there was an order to everything, so it was just a matter of memorizing that order while taking an exam and doing it in my head”* (Student 7).

Another student described the choreography as creating a “movement mnemonic.” *“It kind of gave me the same feeling as remembering a mnemonic for anatomy things where you could associate a certain movement with a certain myotome or dermatome”* (Student 28). In this sense, it wasn't so much the sequence of choreography but a single dance move that was linked to an anatomical fact. This also held true for the smaller hand gestures used as choreographed pieces to remember nerves of the brachial plexus. One student mentioned that she will never forget the median nerve based on the “Come to MEdian nerve” movement that she demonstrated while speaking by curling her fingers.

Choreography driven learning appears very similar to the proprioceptive feedback gained from doing the movement; however, these codes differ in a subtle way. The movement itself is the more memorable piece of information that the anatomical detail could then be applied to with the choreography driven code. However, with the proprioceptive code, memory was strengthened by the “feeling” of the muscle movement rather than memorizing the movement itself.

### Repetition is valued

Repetition as a theme presented itself in two different ways. Seven students attributed the repetitiveness of physical activity within the video as being helpful to their learning, while four participants mentioned that they were inspired to watch the videos multiple times, which ultimately led to increased retention of the material. Video format included a preliminary segment that taught or explained the material, followed by a movement piece that reinforced content and quizzed the viewer. Student 3 commented that *“the repetition of the movements and seeing the movements over and over again was very helpful”*. Student 21 remarked *that “She would explain it at the beginning, and then at the end, she has that little quiz. And she does a couple of rounds in between so you kind of get into the groove of it”*. Additionally, Student 24 noted the frequency at which she watched the video *“I was watching it probably every night, doing the little activities. And then the morning of (the exam), just to prime myself, I watched it one more time as I was brushing my teeth and doing all the movements and stuff”*. In this way, the video proved useful in not just learning the content but also served as a quick exam prep.

### Time‐efficiency

Time efficiency was another notable benefit of the videos. Six students observed that the incorporation of physical activity helped them learn more quickly and retain information better. The combination of movement and study allowed them to absorb the material in a more efficient way, making their learning process feel faster and more effective. *“I think pairing the learning with movement is definitely easier way of learning than sitting down… I spent maybe 15‐20 minutes watching the video and then practicing once or twice for another 20 minutes. I've learned all this material in under an hour and if I wasn't doing that, I would have to spend way more time trying to memorize it. So, learning with dancing is definitely way more efficient”* (Student 16).

Five other students linked the efficiency of the videos to the idea of “killing two birds with one stone,” as the videos allowed them to study while also getting a workout. This subtheme highlights the dual benefits of learning and wellness, showing that students appreciated the efficiency of achieving both at the same time. As one student (Student 9) put it, *“incorporating movement with studying was an incentive to kind of do both things at the same time”*. Student 22 was pleased with the discovery. *“You know it's a new concept in terms of utilizing movement while studying…It doesn't have to be that you're just studying, or you're just moving, and having to find ways to do them both. So, it was a nice introduction”*.

### Study break

Despite actively learning while engaged in the videos, six individuals described this time as a break from studying. *“I felt like I was getting up and moving around which felt like a natural break in my study time”* (Student 14). The physical elements felt good, helped wake students up, and recharged them for additional studying. Student 1 shared, *“Physically, it's good to move. It feels good to not be sitting and to get some blood flow elsewhere… You get a good break”*. Student 28 added that, “I felt more energized and more interested in the subject cause rather than staring at a screen, I was actually moving and doing something”.

This finding is not surprising, as many students noted that they already use movement as a means to disengage from academics and clear their head. For many, physical activity assists with their mental health by serving as a stress reliever. Activities such as walking, running, or going to the gym help them relax and recharge. Time constraints, however, often prevent them from doing so as often as they would like. This barrier circles back to the previous theme of the time efficiency that is gained by providing a movement regime while studying. As commented by Student 21, “we don't get to move that often. We have to take time out of our daily routine to go out and do stuff and move. But if its incorporated into your studying, then we're more likely to do it, because then its not taking extra time out of our time.”

### Fun learning environment

Enjoyment of the videos played a key role in creating a positive learning environment and contributing to mental health. “Fun” was by far the most frequently used word to describe the student experience, with 15 different individuals mentioning it 24 times. Student 15 shared, “It's good to feel like you're being productive in studying… it's definitely a fun way to study, and it also gets you moving, which is really good.”

Students described the “fun factor” in terms of laughing, mood improvement, and being silly. Some enjoyed watching and participating in the videos alone, while six students utilized the videos as an opportunity to congregate with classmates and socialize. *“I felt happy. I felt like it was funny. I felt like all my friends thought it was funny. We all were laughing and smiling before, during and after”* (Student 17). Another student commented on the comradery that developed from discussing the videos with her friends even though she watched them on her own. “*They were talking points among our classmates, like we would jokingly do them with each other. So, it felt fun socially to do them”* (Student 6).

The fun learning environment also helped reduce stress by deconstructing complicated subjects into manageable pieces. Student 15 believed the brachial plexus to be a confusing subject. *“I feel like everyone was really stressed out about it [the brachial plexus]. And then the dancing videos were pretty silly and cute and made you giggle. They made it more fun for sure. It's like this is a silly little thing that you don't need to be stressed about.”* Student 19 also commented that *“I feel like it cleared up some confusion I had, because when I was first looking at the nerves, it felt pretty overwhelming. But when you do a 12‐minute video that is really just doing exercise and associated with the nerves, it kind of helped make it feel a little less daunting.”*

## DISCUSSION

This research aligns with previous studies highlighting the benefits of physically active rest breaks and movement‐based learning. Concentration, alertness, and enjoyment during university classes have all been shown to increase with the introduction of movement breaks in the classroom.^13^, ^14^ Peiris et al.^13^ explained this phenomenon using the effort‐recovery model. The model suggests that regular breaks and relaxation strategies—such as physical activity, mindfulness, or leisure—are essential for maintaining productivity and preventing the negative effects of prolonged exertion.^30^ This theory mirrors some of the perceptions expressed in our study, where the videos created a fun, low‐stress learning environment that allowed students to relax and recharge before engaging with additional content.

The study by Peiris et al.^13^ also supports our findings that the physical activity within the videos promoted social connections among students. Contrary to our research, however, they described fewer benefits among those breaks in which students moved while listening to content. While we did not offer non‐content‐based movement sessions for comparison, our results indicate that when physical activity and learning are combined appropriately, a break‐like atmosphere that helps to re‐energize students for additional studying is still attainable.

Also comparable to this medical student study are the “in‐person” yoga and Pilates anatomy teaching sessions conducted by McCulloch et al.^23^ and Sugrue et al.^24^ Many of the learning benefits identified in their research are echoed in the findings here. Participants described the sessions in overwhelmingly positive terms, particularly in relation to improving their physical and mental well‐being. Much like our video‐based learning, terms such as fun, laughter, relaxation, and stress‐free learning were frequently used to describe the experience. Additionally, participants appreciated that they could spend time focusing on their personal well‐being without feeling guilty for taking time away from their studies. This mirrors our time‐efficient learning theme.

Sugrue et al.^24^ introduced the theory of experiential learning to describe the cognitive benefits of integrating movement with anatomy education. Experiential learning is a process through which individuals learn by actively engaging in an activity, reflecting on the experience, and applying the insights gained to future situations.^31^ In their study, Sugrue et al.^24^ argued that the Yoga Anatomy workshops provided students with an opportunity to reinforce their anatomical knowledge by physically experiencing the application of that knowledge within their own bodies.

While the experiential learning model effectively explains many of the cognitive gains described in this study, we propose that the theory of embodied cognition can also be applied to the data. Embodied cognition is a theory that suggests that our thinking, perception, and understanding are deeply influenced by our physical bodies and the sensory experiences we have.^18^, ^19^, ^20^ One of its key tenets supports the idea that learning is enhanced when we engage in physical activities that connect us to the material. The sensorimotor experience leads to a richer and more elaborate representation of the learning event, and this higher‐quality mental representation results in faster and more effective memory performance.

Sensory input came in many forms while participating in the videos. Similar to the “in‐person” sessions, students could feel the sensation of actively engaged muscles when discussing relevant anatomy that helped solidify that knowledge. Unique to the video experience, however, was the addition of auditory and tactile sensations. Students commented on the tangible benefit that touching a patch of skin had on memory when discussing cutaneous innervation. Tactile sensation was used during exercise to create a richer sensorimotor experience to again embody neuromuscular content. As an unexpected finding, auditory inclusion also enhanced the sensorial experience. Use of popular songs not only created a fun atmosphere but also helped to layer the music to the movement to the knowledge. It is postulated that the well‐known song became linked to anatomy, which resulted in easier retrieval of the material. To the author's knowledge, the additional tactile and auditory strategies were not employed within the yoga anatomy programs.

The theory of embodied cognition also highlights the learning benefits of incorporating gestures. Research suggests that gesturing provides an additional memory code^19^, ^20^, ^32^ that strengthens memory traces and offers extra retrieval cues to aid recall.

In the videos, small hand gestures were choreographed into the brachial plexus dance to represent the functions of the ulnar, median, and anterior interosseous nerves. These included forming the intrinsic plus sign and abducting the fingers to illustrate the ulnar nerve's function. Similarly, curling one's fingers in a “come here” motion, reinforced by the phrase “come to median nerve,” helped elucidate the function of the median nerve, while forming the “OK” sign helped learners remember innervation of the anterior interosseous nerve.

Many of the gestures in these videos were designed to embody cognition by directly linking movements to the motor functions of these nerves. In contrast, the yoga‐based teaching sessions likely focused on poses rather than incorporating gestures as a learning tool, though specific movements were not described.

One final advantage of the movement‐based videos lies in the benefit of repetition. Unlike a single live session, videos allow students to revisit the material multiple times, reinforcing memory through repeated exposure. Additionally, students can review the content right before an exam, helping to bring key concepts to the forefront of their minds. In contrast, the in‐person sessions were a one‐time experience, limiting opportunities for reinforcement.

One drawback of the video sessions, compared to in‐person events, is that students may choose to watch rather than actively participate in the presented physical activity. However, even passive viewing can still provide learning benefits. One student, for example, admitted to being an “armchair participant,” engaging only in the hand movements while remaining seated. While this approach still allows for the cognitive benefits of gesturing, it does not provide the additional wellness benefits associated with full‐body movement.

The myotome dermatome dance offered within one of the videos is not a unique concept, as a quick Google search reveals numerous similar videos. However, what distinguishes the video within this series is its structured teaching approach—integrating explanations of the content alongside the dance moves associated with various spinal levels. In contrast, most YouTube videos jump straight into complex choreography, making it difficult for the average student to follow along.

### Recommendations

Based on the learning benefits presented in this paper and supported by previous research, movement‐integrated anatomy teaching is an effective tool for reinforcing knowledge, improving memory, and promoting student wellness. Faculty interested in leveraging these benefits are encouraged to design their own sessions or take advantage of the Knockout anatomy series of videos, if they feel uncomfortable leading the physical activity themselves.

While this teaching method offers significant advantages, it may not be suitable for everyone. Not all students enjoy movement, and some may have physical limitations that prevent full participation. For this reason, we recommend using movement‐based sessions as a supplemental resource rather than a primary teaching method.

### Limitations

This study had several limitations. First, selection bias toward individuals who enjoy physical activity or active learning is likely present within this research. Individuals who dislike the concept of movement‐centered learning were unlikely to watch the supplemental videos. Not all the participants considered themselves to be active at the time of the interview; however, they all indicated that physicality was important to them in terms of mental and physical health, energy levels, focus, and productivity. Because there was no tracking of which students watched the videos, and there was monetary gain from participating in the interview, it is also possible that a student falsely self‐reported as watching the videos.

Second, the lead researcher and creator of the videos is also an active participant in physical activity and deeply values its role in overall wellness. The themes presented in this paper are likely shaped not only by student perspectives but also by her own beliefs about the significance of exercise.

And lastly, a bias toward female participation is present in this research as evidenced by 18 of the 28 participants. The males that did participate reported similarly to the females in terms of the videos' benefits toward learning.

## CONCLUSION

This study reinforces the benefits of movement‐integrated learning in anatomy education, demonstrating its perceived effectiveness in improving knowledge retention and promoting overall well‐being. By incorporating physical activity into study sessions, students reported stronger connections with the material, increased recall during exams, and a more enjoyable learning experience.

Video‐based learning offers several advantages over previously reported live yoga anatomy teaching sessions, including the ability for students to revisit content multiple times and increased faculty accessibility. Ready‐made videos enable instructors who may not feel confident leading movement sessions to incorporate movement‐based teaching into their curriculum. Additionally, the multi‐modal physical activity featured in these videos incorporates the use of gesturing and tactile sensations to enhance cognitive gains in ways that classic yoga poses may not. However, student independence in video‐based learning may reduce physical participation, potentially limiting wellness benefits. Furthermore, movement‐based learning may not be suitable for all students, particularly those with physical limitations or a preference for more traditional study methods.

Given these findings, movement‐integrated anatomy teaching should be viewed as a valuable supplemental resource rather than a replacement for conventional teaching methods. Faculty members are encouraged to explore and implement movement‐based strategies, whether through self‐designed in‐person sessions or by utilizing resources like the Knockout Anatomy video series.

## AUTHOR CONTRIBUTIONS

**Maureen Schaefer:** Conceptualization; funding acquisition; writing – original draft; investigation; writing – review and editing; data curation; software; formal analysis; methodology; supervision; validation. **Libby Bradley:** Formal analysis; writing – review and editing; writing – original draft; validation; methodology. **Nicole Geske:** Writing – original draft; formal analysis. **Nebiyat Girma:** Data curation; writing – review and editing. **Laura Gjidoda:** Data curation.
